# Reasons for and against Nutritional Interventions. An Exploration in the Nursing Home Setting

**DOI:** 10.3390/geriatrics6030090

**Published:** 2021-09-16

**Authors:** Franz J. Grosshauser, Eva Kiesswetter, Gabriel Torbahn, Cornel C. Sieber, Dorothee Volkert

**Affiliations:** 1 Institute for Biomedicine of Aging, Friedrich-Alexander-Universität Erlangen-Nürnberg, 90408 Nuremberg, Germany; eva.kiesswetter@fau.de (E.K.); gabriel.torbahn@fau.de (G.T.); cornel.sieber@fau.de (C.C.S.); dorothee.volkert@fau.de (D.V.); 2 Department of Medicine, Kantonsspital Winterthur, 8401 Winterthur, Switzerland

**Keywords:** malnutrition, nursing home, nutritional intervention, aged, nurse, awareness

## Abstract

Malnutrition (MN) is widespread in nursing homes. Sometimes, but not always, nutritional interventions (NIs) are made, and the reasons for or against NIs are unknown. The aim of this cross-sectional study was to describe these reasons for residents with and without MN according to nurses’ subjective judgement and according to objective signs of MN. The nutritional status of 246 nursing home residents was subjectively judged by nurses (MN, at risk of MN, no MN) and objectively assessed by body mass index (BMI), weight loss (WL), and low food intake. NIs (enriched meals and/or oral nutritional supplements) were recorded using a standardized questionnaire, and nurses’ main reasons for (not) giving NIs were obtained in an open question. Of the residents, 11.0% were subjectively malnourished, and 25.6% were at risk of MN; 32.9% were malnourished according to objective criteria. Overall, 29.7% of the residents received NIs, 70.4% of those with MN as assessed by the nurses, 53.0% of those with objective MN, and 11.0% and 18.0% of non-malnourished residents, respectively. Reasons for NIs most often stated were low intake (47.9%), WL (23.3%), and low BMI (13.7%). Reasons against NIs mostly mentioned were adequate BMI (32.9%) and sufficient intake (24.3%). The lack of NIs for residents with MN was partially—but not always—explained by valid reasons. As residents without MN frequently received NIs, criteria for both MN rating and providing NIs, require closer scrutiny.

## 1. Introduction

Malnutrition (MN) is a frequent and relevant problem among older adults [1,2], which can adversely affect every organ system and function of the human body [3,4]. It is associated with an increased risk of mortality and morbidity [5], functional decline, hospital stay [3,6], and increased health care costs [7].

In nursing homes (NH), depending on the method used to assess MN, prevalence rates of up to 66% have been reported [8,9,10]. Different parameters are used singly or in combination to define MN, with low BMI, unintended weight loss (WL) and low food intake as the most widespread. In the nutritionDay (nDay) project, a huge international study of more than 20,000 NH residents, a low BMI was reported in 16% of the participants, WL in the previous year in 33% and low intake in 36% [11]. In contrast to these objective criteria, nurses subjectively categorized only about 10% of the residents as malnourished [11,12].

To avoid severe consequences of MN, adequate intervention strategies are crucial. The primary recommendation for older persons who are malnourished or at risk of MN is to offer energy and/or protein-enriched food, followed by oral nutritional supplements (ONS) [1]. In NH residents, too, the positive effects of these nutritional interventions (NIs) are well documented [13,14,15].

Regarding the implementations of nutritional strategies, nurses play an essential role; moreover, they are also essential for detecting malnourished and vulnerable residents [16,17]. In everyday clinical practice, both fortified food and ONS are usually recommended and implemented by nurses. Thus, recent analyses of the nDay in NH project have shown that residents who were malnourished or at risk of MN according to nurses were 3–4 times more likely to receive ONS than those judged to be well-nourished, whereas the chance of receiving ONS based on objective MN criteria was only about twofold. In Germany, prescribing ONS ultimately depends on the attending physician, who may, however, disagree with the nurse. The offer of an enriched diet, on the other hand, depends on the local nursing home policy but is common in German NHs and usually initiated by the nurses. On nDay 2018, 21% of 1551 participating German residents received an enriched diet and 11% ONS [18]. nDay evaluations also revealed that ONS are, however, not used exclusively for malnourished residents, and by no means do all malnourished residents receive ONS, whether judged subjectively or objectively [11].

Thus, questions arise as to why residents with MN often do not receive NIs, whether all malnourished residents need NIs and conversely, if it might be reasonable to provide NIs to non-malnourished residents. The nurses’ reasons for or against NIs are of great interest in order to understand nurses’ attitudes and motives better and to develop targeted education material.

To the authors’ knowledge, little is known about reasons for or against the provision of NIs in the NH setting. Asked about ONS prescription, staff members of Canadian long-term care facilities reported many different reasons, including decreased intake, unintentional WL, and wound healing [19]. So far, studies about reasons for providing other NIs, such as enriched food, are lacking.

Thus, the aim of this exploratory study was to describe the nurses’ main reasons for and against the provision of NIs for residents with and without MN according to nurses’ subjective judgement and according to objective signs of MN.

## 2. Materials and Methods

### 2.1. Study Design

This cross-sectional study was performed within the frame of the nDay project from November 2018 until January 2019. nDay is a worldwide project which aims to improve awareness of MN in hospitals and NHs. It is a one-day annual evaluation using standardized questionnaires (https://www.nutritionday.org, accessed on 25 August 2021).

Twelve randomly selected NHs in the south of Nuremberg (Germany) were contacted by phone, and the project was presented; seven of them agreed to participate. Both baseline nDay questionnaires for the NH setting—one on unit structure and one on residents’ characteristics, nutritional status, and NI—were completed in seven NHs (3 privately run, 3 non-statutory social welfare organizations, 1 welfare health centre), with a median of 69 beds (range 10–104). All nDay questions, and an additional question regarding reasons for or against NIs, were answered in personal interviews with 16 nurses (all female, 12 nursing managers, 4 qualified nurses), one nurse in each participating ward, who was very familiar with all residents on this ward and had between 2 and 18 years of experience. All 16 interviews were conducted by the first author on a one-to-one basis on the respective ward without any disturbance from colleagues or managers. A nutritionist or a dietician was not available in any of these NHs.

The study was approved by the Ethical Committee of the Friedrich-Alexander-University Erlangen-Nürnberg, Germany (309_16 B). Written or oral informed consent was given by the residents or their legal custodians prior to participation.

### 2.2. Participants

At the time of investigation, 405 residents were living in the respective NHs in 22 units, of which 16 were selected by the NH managers for participation. Of 308 residents in these units, 287 (93.2%) residents or their legal representatives agreed to participate. For this analysis, participants with tube feeding or parenteral nutrition (*n* = 7) and residents with a lack of information on WL in the previous three months (*n* = 34) were excluded, resulting in 246 residents.

### 2.3. Residents’ Characteristics

Information on the year of birth, sex, height, cognitive status (severe, mild, no dementia), mobility (bedridden or chair bound, able to go around in the unit, go out of the unit), dysphagia (yes, no), mealtime assistance (yes, no), depression (severe, mild, no), and the number of drugs currently taken per day was gained using the nDay questionnaire with the above-mentioned predefined answer categories (https://www.nutritionday.org/cms/upload/pdf/3_for_nursing_homes/1.3.participate/English_pounds_inches/NH_sheet2_pounds_and_inches_english.pdf, accessed on 25 August 2021). The questionnaires were completed in personal interviews with the nurses using information from the care documentation, including diagnoses by the attending physicians (e.g., regarding dementia and depression). Residents were not involved in the data acquisition process.

### 2.4. Nutritional Status

In the nDay questionnaire, nurses were also asked to rate the presence of MN (yes, at risk, no), referred to as subjective judgement in the remaining text.

On NH admission, BMI was calculated using actual body weight in kilograms divided by body height in meters squared. On nDay, body weight was measured on the nearest 0.1 kg by the nurses using calibrated scales available in the NHs. A cut-off value of <20 kg/m^2^ was used to define a low BMI [2]. Percentage WL was calculated based on body weight on nDay and the weight documented in the resident’s file 3 months earlier. A threshold of ≥5% in the previous 3 months was used to define a significant WL [20]. Food intake at lunch on nDay was rated as 3/4 or all, 1/2, 1/4, or nothing by using plate diagrams. Low food intake was defined as a quarter or nothing. To minimize variation, the same persons (one nurse for every ward) completed all the plate diagrams. Residents were considered to be “objectively malnourished” when at least one of the three objective criteria, BMI, weight loss, or low intake, was present [20].

### 2.5. Nutritional Interventions

As part of the nDay-questionnaire, the nurses were asked if the resident received an enriched diet (yes, no) and if he/she received ONS (yes, no).

### 2.6. Reasons for or against Nutritional Interventions

An additional question with five open answer fields was asked about the reasons for giving or not giving NIs. All reasons mentioned were documented, but mostly only one reason was given. The results reported here are restricted to the main reason for each resident, which were categorized by the first author (FG). Categories and assignments of main reasons were checked and approved by the senior author (DV). After discussion, both authors confirmed the final version.

### 2.7. Data Analyses

Results are reported as mean ± standard deviation (SD) or median with interquartile range (IQR) for continuous variables and absolute and relative frequencies for nominal and categorical variables. Data are presented for the total sample as well as in subgroups according to subjective judgement and objective MN. For all presented variables, complete data is available. To examine if participants were correctly classified as malnourished or well-nourished by the nurses, we calculated sensitivity and specificity based on the cross tables using the objective criteria as the reference standard. For this analysis, persons judged at risk of malnutrition and without malnutrition were summarized in one group. All analyses were performed with SPSS, version 26.0 (IBM, Munich, Germany).

## 3. Results

### 3.1. Residents’ Characteristics and Nutritional Status

The mean age of the participants was 83.6 ± 8.3 years; 67.5% were female. Severe dementia was reported in more than 40%, and severe depression in about one fifth. Twenty-two percent of the residents suffered from dysphagia, and 33.3% required assistance with eating (Table 1).

The nurses considered 11.0% of residents to be malnourished and 25.6% at risk of MN. Residents judged by staff to be malnourished had a mean BMI of 17.7 ± 1.5 kg/m^2^ (Table A1). They were older, more often female, severely demented and immobile, but took fewer drugs than residents without MN.

According to the objective criteria, 32.9% were malnourished. These residents were also older, more often female, severely demented and immobile than residents without objective signs of MN (Table 1).

All 27 residents who were malnourished according to the subjective assessment were also malnourished according to the objective criteria. No resident, who was well-nourished based on the objective criteria, was judged by nurses to be malnourished. On the other hand, one quarter (25.9%) of those with objective signs of MN were judged by nurses as not malnourished, and nearly half (47.6%) of those subjectively judged to be at risk of MN had no objective sign of MN (Table A2). Of the objectively malnourished residents not supported with NI, 37% had a BMI ≥ 22 kg/m^2^, and 29% had a BMI ≥ 24 kg/m^2^. The sensitivity to correctly classify residents as malnourished by the nurses when using the objective criteria as reference standard was 33.3%, the specificity to correctly classify residents without malnutrition was 100%.

### 3.2. Nutritional Interventions

**Figure 1:**
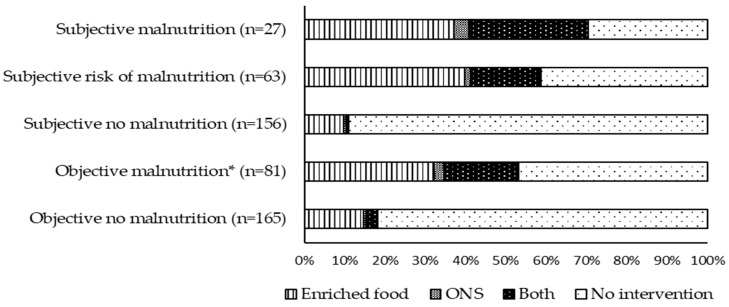
Proportion (%) of residents receiving nutritional interventions stratified by subjective judgement of malnutrition by nursing staff and objective rating of malnutrition (* BMI < 20 kg/m^2^ or weight loss ≥ 5% in last 3 months or intake ≤ 1/4 of the meal).

Seventy-three (29.7%) residents received NIs, 68.5% of them enriched meals, 4.1% ONS, and 27.4% both. Figure 1 presents the provision of NIs stratified by subjective and objective MN. A total of 70.4% of those who were judged by the nurses to be malnourished and 53.0% of residents who were malnourished based on the objective criteria received NIs. A total of 10.9% of subjectively and 18.2% of objectively well-nourished residents received NIs.

### 3.3. Reasons for Nutritional Interventions

The main reason for NIs (*n* = 73) was low intake (47.9%), followed by WL (23.3%), and low BMI (13.7%). A tumor was stated in 8.2% and poor nutritional status in 2.7% as a reason for NIs. In the case of three residents (4.2%), nurses were unable to state a specific reason.

**Figure 2:**
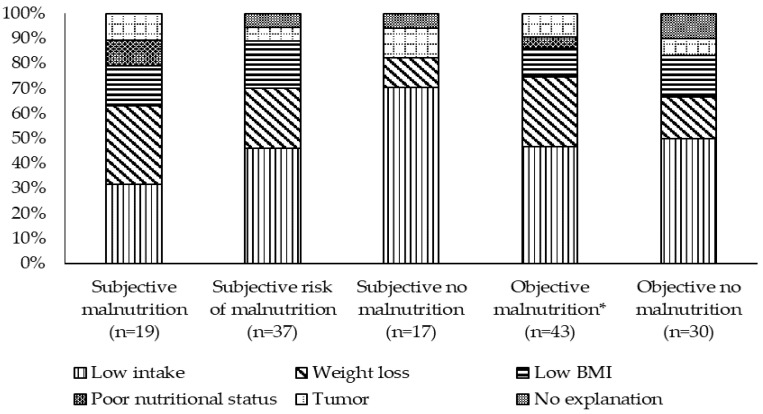
Reasons for nutritional interventions (%) stratified by subjective judgement of malnutrition by nursing staff and objective rating of malnutrition (* BMI < 20 kg/m^2^ or weight loss ≥ 5% in last 3 months or intake ≤ 1/4 of the meal).

The reasons for giving NIs according to both subjective and objective ratings of MN are presented in Figure 2. In 12 of 17 residents judged by nurses as not malnourished, low intake was given as the main reason, while low BMI was not mentioned at all in this subgroup.

### 3.4. Reasons against Nutritional Interventions

The most frequent reason against NIs was an adequate BMI in 32.9% of those residents without NIs (*n* = 173), followed by sufficient intake (24.3%), satisfactory weight (12.7%), awaiting further weight trends (12.1%), and no WL (8.1%). Eight residents (4.6%) were not given NIs because of a living will, five (2.9%) were “not judged as malnourished”, one (0.6%) because of refusal by the legal guardian, one (0.6%) because of a terminal situation, one (0.6) because the staff were awaiting the results of dietary record and in one case (0.6%), no explanation could be given.

**Figure 3:**
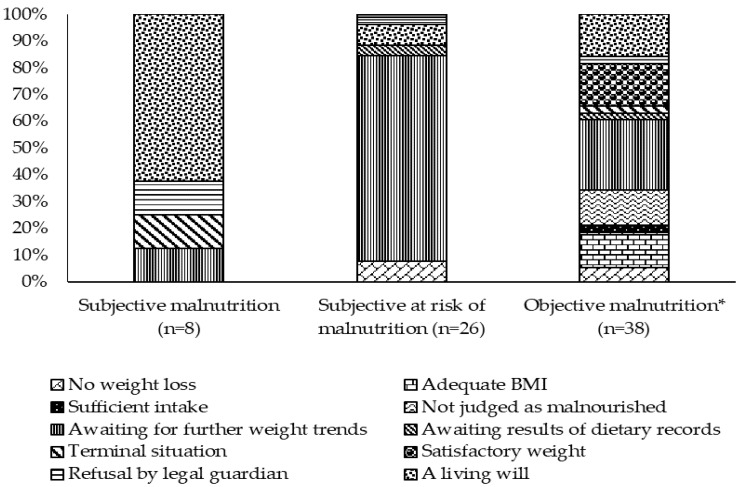
Reasons against nutritional interventions (%) despite (risk of) malnutrition according to subjective rating of malnutrition by nursing staff and objective rating of malnutrition (* BMI < 20 kg/m^2^ or weight loss ≥ 5% in last 3 months or intake ≤ 1/4 of the meal).

Reasons against NIs for residents with MN or at risk of MN are presented in Figure 3. Whereas in residents subjectively judged to be malnourished, a living will was the main reason (62.5%), those at risk of MN did not receive NIs mainly because nurses were awaiting further weight trends (77.0%). Thirty-eight of the residents with objective signs of MN did not receive NIs for a variety of reasons (Figure 3). All these 38 residents presented single objective signs of MN (13 low BMI, 9 WL, 16 low intake) (Table 2).

## 4. Discussion

Looking at nurses’ view of their residents’ need for NIs, we found that nurses generally reported valid reasons for and against NIs. The reasons against NIs for objectively malnourished residents were, however, very diverse and must be questioned in some cases.

### 4.1. Nutritional Interventions

About one-third of all residents received NIs, which is consistent with previous findings in German [18] and French nursing homes [21], and also worldwide [11]. Interestingly, many more residents judged by the nurses to be malnourished than objectively malnourished residents received NI (Figure 1). This underlines the important role of the nurses in nutritional care and is in line with the previous observation of an increased chance of receiving ONS if nurses consider a resident to be malnourished [11]. Further, this implies the significance of nutritional education in the training of nurses as they need the competence to identify those in need of NIs correctly.

Remarkably, non-malnourished residents, according to both subjective and objective ratings, were often also supported with NIs. This support has also been reported in hospital patients [18] and may be a reasonable method in order to prevent MN.

### 4.2. Reasons for Nutritional Interventions

Interestingly, the most frequent reasons for NIs are in accordance with our objective MN criteria, low BMI, WL and low intake. Thus, nurses seem to be basically informed about indicators of malnutrition and indication of NIs. However, given the discrepancy between subjective and objective malnutrition ratings, the exact criteria for their judgement seem to be at least partially different from the objective criteria.

Decreased intake and unintentional WL were also the main reasons reported by staff members of Canadian long-term care facilities for prescribing ONS. There, in addition to wound healing, pressure ulcers and swallowing difficulties were named [19], which are rather indirect indications for NIs probably because of low intake in these persons, and were not mentioned in our study. About one-fifth of our residents had dysphagia (Table 1); nevertheless, dysphagia was not cited as a reason for NIs. Conversely, “poor nutritional status” was stated as an additional reason for NIs. Unfortunately, the precise meaning of this term remains unclear.

### 4.3. Reasons against Nutritional Interventions

The reasons for not giving NIs to the eight residents with subjective MN were in seven cases reasonable: a living will, refusal by the legal guardian, and a terminal situation (Figure 3). Awaiting further weight trends in a malnourished person is, however, questionable since immediate measures are required in the case of MN to prevent further deterioration [1]. In more than 80% of the residents judged to be at risk of MN and receiving no NI, “wait and see” is the major strategy (Figure 3). In these residents, it cannot be excluded that after updated parameters (i.e., body weight and nutritional intake) were checked, NI was initiated. “Wait and see” was also the most frequently cited reason for no NI in objectively malnourished residents, in addition to the valid reasons mentioned above (Figure 3, Table 2). Of the remaining 19 residents (lower half of Table 2), 10 with low food intake on nDay did not receive NIs. Here, it could be that the amount of food eaten on nDay does not reflect usual intake and may be the result of exceptionally poor appetite or dislike of the meal served. Four residents with low BMI did not receive a NI because they were not judged to be malnourished. Therefore, it cannot be ruled out that these residents have always been petite persons with lifelong low BMI. In two residents with WL, nurses seemed to be unaware of this problem. Two other residents with WL did not receive NIs, because of “adequate BMI”. All residents not supported with NI and judged by the nurses to have adequate BMI had BMI-values greater than 20 kg/m^2^. In these cases, BMI seems to be more relevant to the decision and WL is not recognized as a relevant indication. Moreover, not all persons who have experienced WL may need a NI, e.g., in the case of a currently improved appetite or an intake that is now deemed sufficient.

### 4.4. Objective Malnutrition Criteria

We considered a BMI below 20 kg/m^2^ and an unintentional WL of ≥5 kg within the previous three months as the objective and reproducible markers of MN, which are widely used and are generally accepted MN criteria in older persons [1,9,20,22]. In addition, we considered low food intake, an important aetiological sign of MN, known as a strong predictor of six-month mortality [23]. Each of these three objective criteria may indicate malnutrition and justify NIs, but does not necessarily have to lead to these measures, e.g., in an end-of-life situation [24,25]. The selection of these criteria is also based on the results of the “Malnutrition in the elderly” Knowledge Hub. In this project, different combinations of diagnostic criteria were compared (low BMI, weight loss, decreased food intake). One recommendation of this project was to look at each criterion separately as each of them may indicate malnutrition [26]. For greater clarity, we preferred the use of these single objective criteria to the widely used MNA or other screening tools, which combine several aspects and thus obscure the meaning of each individual variable. We are aware of the limitations of BMI as well as low food intake as sole markers of malnutrition but were also interested in the reasons against NIs in the case of low BMI or low intake.

### 4.5. Recognition of MN by Nurses

We observed a discrepancy between subjective judgement and objective criteria of MN. Nurses’ evaluations were highly specific but very insensitive to objective MN criteria. At first sight, nurses might not have recognized MN properly. However, out of the 54 residents with objective signs but not considered malnourished by the nurses, 33 were categorized as at risk of MN (Table A2), which indicates some kind of awareness. Nevertheless, objective signs were not recognized in more than one-quarter of the residents. A reason for underestimating MN could be that MN may be masked by a high BMI, as BMI is often used as a single parameter for nutritional assessment [27]. Of the objectively malnourished residents not supported with NI, 37% had a BMI ≥ 22 kg/m^2^ and 29% had a BMI ≥ 24 kg/m^2^. This may indicate that nurses believe residents are of normal weight and do not identify MN in the case of WL or poor intake [28]. This assumption is supported by the fact that objectively malnourished residents not supported with NI very often had high BMI (37% ≥ 22 kg/m^2^ and 29% ≥ 24 kg/m^2^. This assumption is also supported by the very low mean BMI of 17.7 kg/m^2^ and more than 90% BMI < 20 kg/m^2^ in subjectively malnourished residents (Table 2). Unfortunately, the exact criteria for and appropriateness of nurses’ classification cannot be clarified definitely.

### 4.6. Strengths and Weaknesses of the Study

A certain strength of this study is that it has made available the main reasons for and against NIs in a large sample of residents from 16 NH units from the perspective of the nursing staff. All nurses interviewed were very experienced and familiar with their residents; thus, it can be assumed that the information is reliable. We focussed our study on these reasons in relation to subjective and objective malnutrition criteria assuming relevant discrepancies that have been confirmed.

We acknowledge that our study has some limitations. Thus, our definition of objective MN, despite using generally accepted criteria, turned out to be debatable. The threshold for malnutrition can differ, according to both BMI (depending on age) and weight loss (e.g., 3, 6 or 12 months) values. Perhaps the amount of food consumed can only be properly evaluated when observed over several days, or other parameters such as loss of muscle mass or signs of inflammation need to be considered in defining malnutrition. We decided to use simple single criteria [12,29] because more comprehensive approaches, such as the application of the Global Leadership Initiative on Malnutrition (GLIM) criteria [30] and subsequent individual assessment, were unfortunately not possible for practical reasons and restrictions. A selection bias cannot be ruled out as not all contacted NHs agreed to participate, and units were pre-selected by the NH management. Nurses and residents from other units and also from other NHs might have altered the results. As health care and educational system differ, we cannot generalize our findings and apply them to other countries. Moreover, our results could be affected by the special situation in Germany, where oral nutritional supplements may not be prescribed by nurses themselves, and it is not known for what reasons the NIs were originally prescribed and by whom. Finally, the questionnaire used has not been validated, bearing the risk of misleading results.

### 4.7. Implications

As nurses usually know the NH residents better than the doctors, they may recognize changes in nutritional status and needs for nutritional support earlier. Therefore, great emphasis should be placed on nutritional aspects in basic and advanced training of nurses with regards to detecting malnutrition and providing nutritional care. With better nutritional training, it should be discussed whether licensed practical nurses and registered nurses should be allowed to prescribe nutritional interventions independent of physicians, as is practiced in other countries [31].

## 5. Conclusions

This explorative study highlights nurses’ views on and reasons for and against NIs in a large sample of NH residents with and without MN according to subjective and objective criteria and reveals interesting discrepancies which need further, more detailed investigation. The lack of NIs for residents with (risk of) MN was partially—but not always—explained by valid reasons. As residents without (at risk of) MN frequently received NI, criteria for both subjective as well as objective MN ratings, as well as criteria for providing NIs, require closer scrutiny. Longitudinal studies investigating which NH residents benefit from the provided NIs, based on the subjective reasons for provision and on objective criteria, could contribute to a more differentiated understanding of this topic.

## Figures and Tables

**Table 1:** Residents’ characteristics in the total sample, according to subjective judgement of malnutrition by nursing staff and according to objective rating * of malnutrition (MN).

			Subjective Judgement			Objective Criteria	
		Total	MN	At risk	No MN	MN	No MN
		*n* = 246	*n* = 27	*n* = 63	*n* = 156	*n* = 81	*n* = 165
Gender, female, *n* (%)		166 (67.5)	22 (81.4)	38 (60.3)	106 (67.9)	63 (77.8)	103 (62.4)
Age (years), mean ± SD (95% CI)		83.6 ± 8.3 (82.6–84.7)	87.5 ± 7.4 (84.5–90.4)	84.6 ± 8.4 (82.5–86.8)	82.5 ± 8.1 (81.2–83.8)	85.3 ± 8.2 (83.4–87.1)	82.8 ± 8.2 (81.5–84.1)
BMI [kg/m^2^], <20		37 (15.0)	25 (67.6)	7 (18.9)	5 (13.5)	37 (100.0)	0 (0.0)
Weight loss, ≥5% within 3 months		30 (12.2)	7 (23.3)	20 (66.7)	3 (10.0)	30 (100.0)	0 (0.0)
Poor intake, ≤1/4 of the meal		38 (15.4)	12 (31.6)	12 (31.6))	14 (36.8)	38 (100.0)	0 (0.0)
Dementia,	Severe	110 (44.7)	17 (63.0)	32 (50.8)	61 (39.1)	41 (50.6)	69 (41.8)
*n* (%)	Mild	98 (39.8)	6 (22.2)	25 (39.7)	67 (42.9)	28 (34.6)	70 (42.4)
	No	38 (15.5)	4 (14.8)	6 (9.5)	28 ((18.0)	12 (14.8)	26 (15.8)
Depression	Severe	56 (22.8)	7 (26.0)	15 (23.8)	34 (21.8)	20 (24.7)	36 (21.8)
*n* (%)	Mild	91 (37.0)	11 (40.7)	18 (28.6)	62 (39.7)	27 (33.3)	64 (38.8)
	No	99 (40.2)	9 (33.3)	30 (47.6)	60 (38.5)	34 (42.0)	65 (39.4)
Number of drugs, median (95% CI)		7 (6.5–7.3)	5 (4.3–6.7)	7 (5.9–7.3)	7 (6.8–7.8)	7 (5.7–7.1)	7 (6.7–7.6)
	Bedridden or	109 (44.3)	18 (66.7)	30 (47.6)	61 (39.1)	39 (48.2)	70 (42.4)
	chairbound						
Mobility	Able to go around	124 (50.4)	8 (29.6)	32 (50.8)	84 (53.8)	39 (48.2)	85 (51.6)
*n* (%)	in the unit						
	Goes out of the unit	13 (5.3)	1 (3.7)	1 (1.6)	11 (7.1)	3 (3.6)	10 (6.0)
Dysphagia *n* (%)		54 (22.0)	14 (51.9)	20 (31.7)	20 (12.8)	26 (32.1)	28 (17.0)
Assistance at the meal (%)		80 (32.5)	19 (70.4)	24 (38.1)	37 (23.7)	36 844.4)	44 (26.7)

* BMI < 20 kg/m^2^ or weight loss ≥ 5% in last 3 months or intake ≤ 1/4 of offered lunch; CI = confidence interval; SD = standard deviation, IQR = interquartile range.

**Table 2:** Reasons against nutritional interventions (NI) stated by nursing staff and presence of objective signs of malnutrition in 38 objectively malnourished residents who did not receive NI. Numbers in () represent number of residents with the respective reason.

	BMI < 20 kg/m^2^	Weight Loss ≥5% in Last 3 Months	Intake ≤1/4 of the Meal
A living will (*n* = 6)	5	0	1
Terminal situation (*n* = 1)	1	0	0
Refusal by legal guardian (*n* = 1)	1	0	0
Awaiting further weight trends (*n* = 10)	1	4	5
Awaiting results of dietary records (*n* = 1)	0	1	0
Sufficient weight (*n* = 6)	0	0	6
Not judged as malnourished (*n* = 5)	4	0	1
Adequate BMI (*n* = 5)	0	2	3
No weight loss (*n* = 2)	0	2	0
Sufficient intake (*n* = 1)	1	0	0

## Data Availability

The data presented in this study are available on request from the corresponding author.

## Appendix A

**Table A1:** Objective criteria for malnutrition * in the total sample, stratified by subjective judgement of malnutrition by nursing staff and by objective rating * of malnutrition (MN).

	Total	Subjective Judgement			Objective Criteria *	
	*n* = 246	MN *n* = 27	At Risk *n* = 63	No MN *n* = 156	MN *n* = 81	No MN *n* = 165
BMI [kg/m^2^], mean ± SD	25.1 ± 5.2	17.7 ± 1.5	23.3 ± 2.5	27.1 ± 5.1	21.3 ± 3.9	27.0 ± 4.8
BMI < 20 kg/m^2^, *n* (%)	37 (15.0)	25 (92.6)	7 (11.1)	5 (3.2)	37 (45.7)	0 (0.0)
Weight loss ≥ 5% in last 3 months, *n* (%)	30 (12.2)	7 (25.9)	20 (31.7)	3 (1.9)	30 (37.0)	0 (0.0)
Intake ≤ 1/4 of the meal, *n* (%)	38 (15.4)	12 (44.4)	12 (19.0)	14 (9.0)	38 (46.9)	0 (0.0)

* BMI < 20 kg/m^2^ or weight loss ≥ 5% in last 3 months or intake ≤ 1/4 of the meal; SD = standard deviation.

**Table A2:** Agreement of subjective judgement of nursing staff and objective rating * of malnutrition (MN).

			Objective Criteria *		
			MN	No MN	Total
	MN	*n*	27	0	27
		row %	(100.0)	(0.0)	(100.0)
		column %	(33.3)	(0.0)	(11.0)
		*n*	33	30	63
Subjective	At risk	row %	(52.4)	(47.6)	(100.0)
judgement		column %	(40.7)	(18.2)	(25.6)
		*n*	21	135	156
	No MN	No MN row %	(13.5)	(86.5)	(100.0)
		Column %	(25.9)	(81.8)	(63.4)
		*n*	165	81	246
Total		row %	(67.1)	(32.9)	(100.0)
		coumn %	(100.0)	(100.0)	(100.0)

* BMI < 20 kg/m^2^ or weight loss ≥ 5% in last 3 months or intake ≤ 1/4 of the meal.
